# An Uncommon Encounter of Roseomonas gilardii Bacteremia: A Case Report

**DOI:** 10.7759/cureus.60667

**Published:** 2024-05-20

**Authors:** Steven Pham, Benjamin Heigle, Prabhneet Pannu, Katie Lee, Connor Gibbs

**Affiliations:** 1 Internal Medicine, Unity Health-White County Medical Center, Searcy, USA; 2 Internal Medicine, Unity Health, Searcy, USA; 3 Graduate Medical Education, Unity Health, Searcy, USA; 4 Emergency Medicine, Unity Health-White County Medical Center, Searcy, USA

**Keywords:** roseomonas gilardii, imipenem, infection, bacteria, roseomonas

## Abstract

*Roseomonas *genus was initially described in 1993 as a "pink coccoid." It is a non-fermentative, aerobic, and gram-negative bacteria. This genus has been uncovered in diverse environmental niches, ranging from water and soil to air and plants. Despite its prevalence in the natural world, human infections stemming from *Roseomonas *species remain a rare occurrence. This organism is also known to be resistant to standard antibiotics. We present a case of an 85-year-old woman with *Roseomonas gilardii *(RG) bacteremia who is a resident at an assisted living facility. Healthcare providers should consider this bacterium in slow-developing gram-negative infections, potentially opting for broad-spectrum antibiotics as an initial treatment.

## Introduction

*Roseomonas gilardii* (RG) is a non-fermentative gram-negative coccobacilli that belongs to the genus *Roseomonas*, found commonly in freshwater lakes or air-conditioning systems [1,2]. It is characterized by the distinctive pink pigmentation [1,3,4]. In 1993, Rihs classified the genus into six distinct *Roseomonas *species utilizing biochemical and DNA hybridization techniques [1]. Among these species, RG is commonly associated with human infections. However, human infections from RG are rare, posing uncertainties regarding its clinical significance. A previous study conducted by Wang et al.'s on 20 patients infected with *Roseomonas *from 2000 to 2010 highlights that catheter-related bloodstream infections are a common presentation [1]. This infection is also often associated with malignancy [1]. While human infections are infrequently reported, *Roseomonas *has been isolated from blood associated with central venous catheters, occasionally linked to peritonitis, cellulitis, septic arthritis, and osteomyelitis [2,5]. *Roseomonas *has the potential to cause infection across diverse age groups, impacting both adults and children regardless of their immune status [1]. However, it is also important to note that the bacterium tends to predominantly affect immunocompromised individuals, further emphasizing its opportunistic nature [1]. Some debilitating factors include but are not limited to chronic kidney disease, diabetes mellitus, ulcerative colitis, and cystic fibrosis [1,3]. The bacteria demonstrates a slow growth pattern, typically requiring four to five days for positive results in blood cultures [1,3]. Furthermore, the growth is limited and often appears in only one out of two blood culture bottles [1,3].

The susceptibility patterns of *Roseomonas *vary. According to the study conducted by Rihs et al., the bacteria was only susceptible to amikacin, gentamycin, and imipenem [6-8]. *Roseomonas *are also found to be resistant to newer-generation cephalosporins, cefotaxime, and ceftazidime [4,8]. Another study reported resistance to trimethoprim/sulfamethoxazole (Bactrim), piperacillin/tazobactam (Zosyn), and ceftazidime [6-8]. Despite primarily affecting immunocompromised hosts, *Roseomonas *infections have a relatively low mortality rate, with most patients achieving complete recovery [1,4,8].

## Case presentation

**Figure 1:**
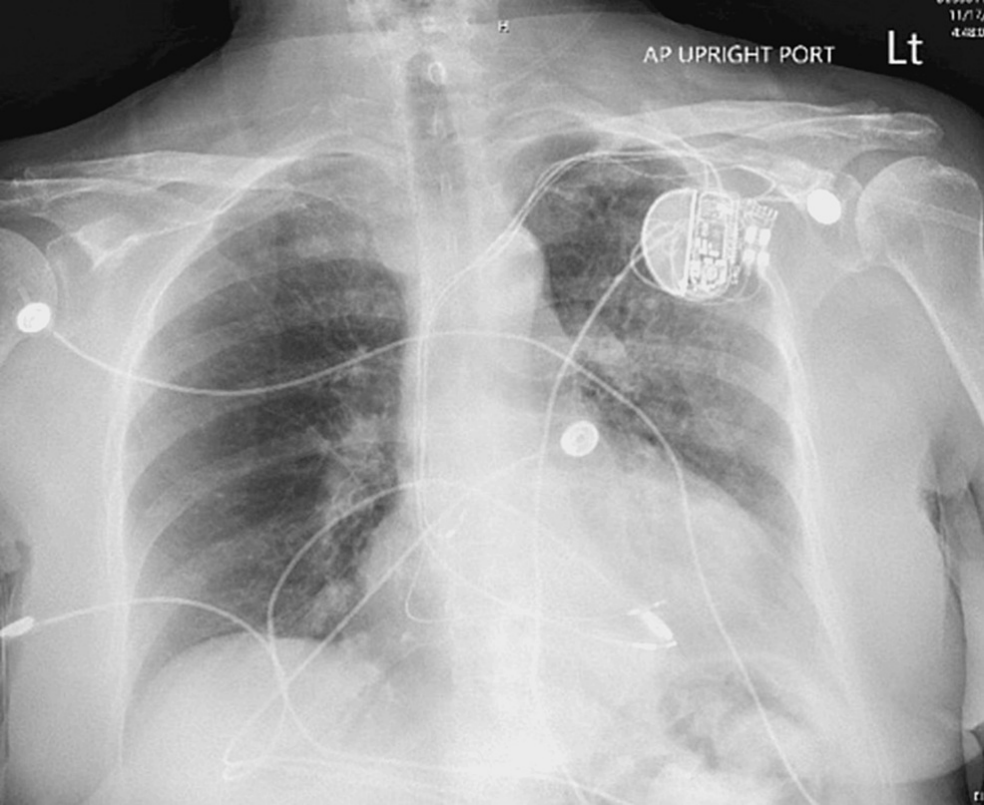
Chest X-ray shows developing pneumonia on the left.

An 85-year-old woman with a past medical history of chronic kidney disease, atrial fibrillation with the presence of a pacemaker, diabetes, hypertension, and Parkinson's disease was admitted from an assisted living facility due to weakness, falls, and breathing problems requiring two liters of oxygen. The patient's daughter reported a history of recurrent urinary tract infections (UTIs). A complete blood count (CBC) showed an increased white blood cell count of 12.7 th/uL, indicating leukocytosis. Chest X-ray imaging suggested possible aspiration pneumonia (Figure 1). Urinalysis suggested a UTI; however, the patient denied having any urinary symptoms. Blood and urine cultures were taken, and the patient was hospitalized for intravenous (IV) antibiotic therapy, receiving two grams of ceftriaxone daily.

**Figure 2:**
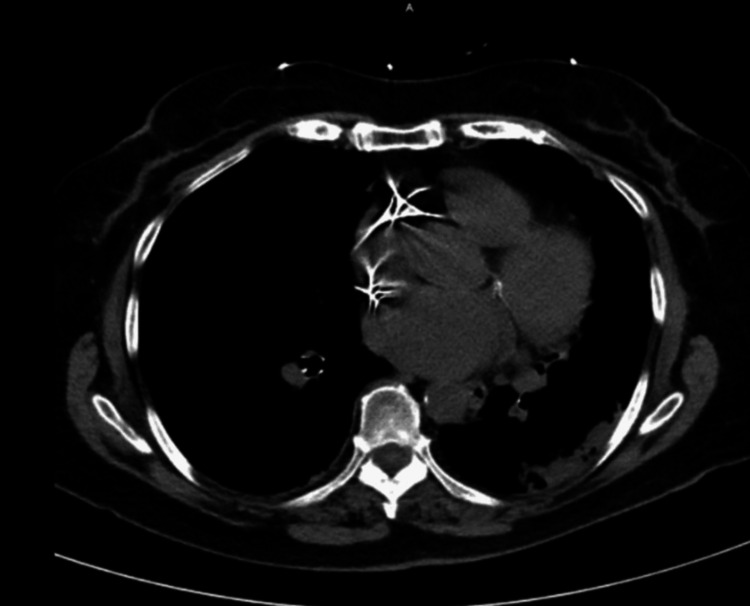
CT chest confirms left lower lobe consolidation, consistent with pneumonia.

On the second day, the patient's ongoing breathing difficulty led to a chest CT scan showing left lower lobe pneumonia and a small left-sided pleural effusion (Figure 2). Treatment involved 500 mg azithromycin for community-acquired pneumonia. Urine cultures detected *Klebsiella pneumoniae*, treatable with ceftriaxone. The patient improved and was discharged to the assisted living facility with oral cefdinir to finish her course of antibiotics.

Six days later, advanced testing (used with specific characteristics such as a lower temperature of 28-30 degrees Celcius and incubation time) found RG in one of the bottles of blood culture. This was positive on day 4 during her hospital course. Due to this finding, the patient was contacted to return to our facility for IV antibiotics. She was given 500 milligrams of imipenem with cilastatin (Primaxin) every eight hours for adequate coverage. Repeat blood culture was negative after 72 hours on both bottles. To continue treatment, a peripherally inserted central catheter (PICC) was placed, enabling discharge to the assisted living facility for ongoing IV antibiotic therapy in stable condition. She completed the therapy for 14 days.

## Discussion

RG infection is rare. Existing literature has indicated that the primary source of RG infection is often bacteremia from the use of a central line [6]. In addition, other infection sites include the respiratory system, wounds or bone infection, peritoneum, enteric regions, and kidney transplant sites, following this specific order of occurrence [6,7,9]. Another feature of RG infection is that it commonly manifests in patients with underlying debility, with 80% of reported cases associated with pre-existing conditions [10-12]. Malignancy emerges as the most prevalent underlying disease, followed by renal disease, inflammatory bowel disease, diabetes, and other factors such as alcohol abuse, cystic fibrosis, and circulatory insufficiency [6].

The antibiotic susceptibility pattern of *Roseomonas *is particularly resistant to cephalosporins. Most *Roseomonas *species exhibit over 96% resistance to cephalosporins, including cefotaxime, ceftriaxone, and ceftazidime [7,8]. However, there is conflicting information, as Lewis reports susceptibility to cefotaxime in her isolates, with at least two patients achieving full recovery on this antibiotic [6]. No data on susceptibility to fourth-generation cephalosporins, such as cefepime and cefpirome, are available. The species universally shows susceptibility to imipenem, amikacin, gentamicin, tobramycin, and tetracycline, but is largely resistant to penicillins, including extended-spectrum penicillins like piperacillin and mezlocillin. Data on susceptibility to newer quinolones is lacking, but Rihs et al. found that 65% of the 42 studied isolates were susceptible to ciprofloxacin, and all other tested isolates showed susceptibility to this antibiotic [6-9].

Despite the association of this infection with debilitated patients, the mortality rate remains relatively low, and patients typically achieve full recovery [6]. Only two deaths are reported in the literature, but both are attributed to underlying diseases, including human immunodeficiency virus (HIV) and chronic lung disease, rather than *Roseomonas *infection itself [7].

We have found several similarities for RG bacteremia in this patient as compared to other literatures. This bacterium tends to thrive in sick individuals with other comorbidities, and our patient presented with weakness and multiple falls along with having chronic kidney disease. Only one blood culture set yielded a positive result, which aligns with existing literature where singular positive cultures are common occurrences [3,6,9]. The positive culture resulted on day 4, which is consistent with documented slow-growth properties. With Primaxin, the patient experienced a complete recovery.

## Conclusions

This case further highlights that RG is a rare opportunistic infection, particularly in individuals with compromised immune systems or with multiple co-morbidities, as in this patient. Further literature reviews show that this infection tends to manifest in individuals with underlying comorbidities, such as malignancy, renal disease, inflammatory bowel disease, diabetes, and many others. The distinct characteristics of RG, such as its propensity for multi-drug resistance and slow growth, should not be overlooked. From past literature, RG seems to be resistant to cephalosporins; therefore, our patient had to be readmitted for imipenem. Prior research has indicated that this organism predominantly flourishes in the presence of a central line, despite the absence of such a line in our patient.

This case report aims to underscore that we should not presume contaminants based solely on one positive blood culture. In cases of gram-negative septicemia in debilitated patients, clinicians should consider RG as a plausible differential diagnosis, particularly in situations involving the presence of a central line. This consideration becomes even more pertinent when blood cultures show no growth for at least three to four days. Despite the occurrence of this infection in debilitated patients, mortality remains relatively low, and patients generally recover completely. Clinical improvement was observed in our patient after imipenem initiation, reinforcing the belief that this organism is mostly treatable.
